# Fatigue Life Prediction of Fiber-Reinforced Ceramic-Matrix Composites with Different Fiber Preforms at Room and Elevated Temperatures

**DOI:** 10.3390/ma9030207

**Published:** 2016-03-17

**Authors:** Longbiao Li

**Affiliations:** College of Civil Aviation, Nanjing University of Aeronautics and Astronautics, No. 29 Yudao St., Nanjing 210016, China; llb451@nuaa.edu.cn; Tel.: +86-25-8489-5963

**Keywords:** ceramic-matrix composites (CMCs), fatigue, life prediction

## Abstract

In this paper, the fatigue life of fiber-reinforced ceramic-matrix composites (CMCs) with different fiber preforms, *i.e.*, unidirectional, cross-ply, 2D (two dimensional), 2.5D and 3D CMCs at room and elevated temperatures in air and oxidative environments, has been predicted using the micromechanics approach. An effective coefficient of the fiber volume fraction along the loading direction (ECFL) was introduced to describe the fiber architecture of preforms. The statistical matrix multicracking model and fracture mechanics interface debonding criterion were used to determine the matrix crack spacing and interface debonded length. Under cyclic fatigue loading, the fiber broken fraction was determined by combining the interface wear model and fiber statistical failure model at room temperature, and interface/fiber oxidation model, interface wear model and fiber statistical failure model at elevated temperatures, based on the assumption that the fiber strength is subjected to two-parameter Weibull distribution and the load carried by broken and intact fibers satisfies the Global Load Sharing (GLS) criterion. When the broken fiber fraction approaches the critical value, the composites fatigue fracture.

## 1. Introduction

Ceramic materials possess high strength and modulus at elevated temperatures. However, their use as structural components is severely limited because of their brittleness. Continuous fiber-reinforced ceramic-matrix composites (CMCs), by incorporating fibers in ceramic matrices, however, can possess higher specific strength, withstand much higher temperatures exceeding the capability of current nickel alloys typically used in the high-pressure turbines, which can lower the fuel burn and emissions, while increasing the efficiency of aero engines [1]. CMC durability has been validated through ground or commercial flight testing in demonstrator or customer gas turbine engines accumulating almost 30,000 h of operation. The SiC/SiC combustion chamber and high-pressure turbine components were designed and tested in the ground testing of GEnx aero engines [2]. The SiC/SiC rotating low-pressure turbine blades in a F414 turbofan demonstrator engine were successfully tested for 500 grueling cycles to validate the unprecedented temperature and durability capabilities by GE Aviation. The C/SiC tail nozzles were designed and fabricated by SNECMA (SAFRAN, Paris, France) and completed the first commercial flight on CFM56-5B aero engine (CFM International, Cincinnati, OH, USA) on 2015. CMCs will play a key role in the performance of CFM’s LEAP turbofan engine (CFM International), which would enter into service in 2016 for Airbus A320 and 2017 for the Boeing 737 max [3].

Upon first loading to fatigue peak stress, matrix multicracking, fiber/matrix interface debonding, and partially fiber fractured in the interface debonded and bonded region, would occur [4,5]. With increasing number of cycles, interface shear stress decreases due to interface wear when fibers slide relative to matrix during unloading and subsequent reloading, which reduces the load transfer capacity between fibers and the matrix [6]. The SiC fiber strength also degrades with increasing number of cycles due to interface wear, which reduces the load carrying capacity of fibers [6]. At elevated temperatures, matrix cracks would serve as avenues for the ingress of environment atmosphere into the composite [7]. When the oxidizing gas ingresses into the composite, a sequence of events is triggered starting first with the oxidation of the interphase, and then fibers. With increasing oxidation time, the oxidation region propagates; and the interface shear stress and fiber strength decrease. Under cyclic fatigue loading, fibers gradually fracture due to the degradation of interface shear stress and fibers strength [8]. When the broken fibers’ fraction approaches the critical value, the composites fatigue fail.

The objective of this paper is to predict the fatigue life of fiber-reinforced CMCs with different fiber preforms, *i.e.*, unidirectional, cross-ply, 2D, 2.5D and 3D CMCs at room and elevated temperatures in air and oxidative environments. An effective coefficient of the fiber volume fraction along the loading direction (ECFL) was introduced to describe the fiber architecture of the preforms. The Budiansky-Hutchinson-Evans shear-lag model was used to describe the micro stress field of the damaged composite considering fiber failure. The statistical matrix multicracking model and fracture mechanics interface debonding criterion were used to determine the matrix crack spacing and interface debonded length. Under cyclic fatigue loading, the fibers’ broken fraction was determined by combining interface wear model and fibers’ statistical failure model at room temperature, and interface/fibers’ oxidation model, interface wear model and fibers’ statistical failure model at elevated temperatures, based on the assumption that the fiber strength is subjected to two-parameter Weibull distribution and the load carried by the broken and intact fibers satisfies the Global Load Sharing (GLS) criterion. The fatigue life and fatigue limit of unidirectional C/SiC, cross-ply C/SiC, 2D C/SiC, 2.5D C/SiC, and 3D C/SiC composites at room and elevated temperatures have been predicted.

## 2. Materials and Experimental Procedures

### 2.1. Unidirectional and Cross-Ply C/SiC Composite

T-700™ carbon (Toray Institute Inc., Tokyo, Japan) fiber-reinforced silicon carbide matrix composites were provided by Shanghai Institute of Ceramics, Shanghai, China [9]. The unidirectional and cross-ply C/SiC composites were manufactured by hot-pressing method. The volume fraction of fibers was approximately 40%. The dog-bone shaped specimens were cut from 150 mm × 150 mm panels by water cutting. The tension-tension fatigue tests at room temperature and 800 °C in air were conducted on a MTS Model 809 servo hydraulic load-frame (MTS Systems Corp., Minneapolis, MN, USA). The fatigue experiments were in a sinusoidal wave form with a loading frequency of 10 Hz. The fatigue load ratio (*σ*_min_/*σ*_max_) was 0.1, and the maximum number of applied cycles was defined to be 1,000,000 cycles. The fatigue tests were conducted under load control in accordance with the procedure in ASTM standard C 1360 [10] at room temperature and 800 °C in air.

### 2.2. 2D C/SiC Composite

T-300™ carbon (Toray Institute Inc.) fiber-reinforced silicon carbide matrix composites were processed by chemical vapor infiltration (CVI) into woven 0°/90° preforms [11]. The volume fraction of fibers was approximately 45%, and the porosity content was about 22%. The dog-bone shaped specimens were cut from 200 mm × 200 mm panels using diamond tooling. The tension-tension fatigue tests at room temperature were conducted on a servohydraulic load-frame that was equipped with edge-loaded grips. The fatigue experiments were performed under load control at a sinusoidal wave form and a loading frequency of 10 Hz. The fatigue load ratio (*σ*_min_/*σ*_max_) was 0.1, and the maximum number of applied cycles was defined to be 1,000,000 cycles.

T-300™ carbon fiber-reinforced silicon carbide matrix composites were manufactured using chemical vapor infiltration (CVI) into woven 0°/90° preforms by Honeywell Advanced Composites Inc., Newark, DE, USA [12]. The volume fraction of fibers was approximately 45%. The dog-bone shaped specimens were cut from 216 mm × 216 mm panels. After machining specimens, they were seal coated with SiC via chemical vapor deposition (CVD). The tension-tension fatigue tests at 550 °C in air were conducted on a servohydraulic load-frame. The fatigue experiments were performed under load control at a sinusoidal wave form and a loading frequency of 10 Hz. The fatigue load ratio (*σ*_min_/*σ*_max_) was 0.05, and the maximum number of applied cycles was defined to be 1,000,000 cycles.

T-300™ carbon fiber-reinforced silicon carbide matrix composites were manufactured using chemical vapor infiltration (CVI) into woven 0°/90° preforms [13]. The volume fraction of fibers was approximately 40%. The dog-bone shaped specimens were cut from the composite panels using diamond tooling. The tension-tension fatigue tests at 1300 °C in the oxidative environment were conducted on a servohydraulic load-frame. The fatigue experiments were performed under load control at a sinusoidal wave form and a loading frequency of 3 Hz. The fatigue load ratio (*σ*_min_/*σ*_max_) was 0.1, and the maximum number of applied cycles was defined to be 100,000 cycles.

### 2.3. 2.5D C/SiC Composite

T-300™ carbon fiber-reinforced silicon carbide matrix composites were manufactured using CVI into 2.5D woven preforms by Shanghai Institute of Ceramics [14]. The volume fraction of fibers was approximately 45%. The dog-bone shaped specimens were cut from 150 mm × 150 mm composite panels using diamond tooling. The tension-tension fatigue tests at room temperature and 800 °C in air were conducted on a MTS Model 809 servo hydraulic load-frame (MTS Systems Corp.). The fatigue experiments were performed under load control at a loading frequency of 10 Hz. The fatigue load ratio (*σ*_min_/*σ*_max_) was 0.1, and the maximum number of applied cycles was defined to be 1,000,000 cycles.

T-300™ carbon fiber-reinforced silicon carbide matrix composites were manufactured using CVI into 2.5D woven preforms [15]. The volume fraction of fibers was approximately 40%, and the porosity content was about 17%. The dog-bone shaped specimens were cut from composite panels using the diamond tooling, and then coated with a SiC coating of about 50 μm in thickness. The tension-tension fatigue tests at 900 °C in air were conducted on a servohydraulic mechanical testing machine (FTM-HT, Strength Research Institute of the Academy of Science, Kiev, Ukraine). The fatigue experiments were performed under load control at a loading frequency of 15 Hz. The fatigue load ratio (*σ*_min_/*σ*_max_) was 0.1, and the maximum number of applied cycles was defined to be 1,000,000 cycles.

### 2.4. 3D C/SiC Composite

T-300™ carbon fiber-reinforced silicon carbide matrix composites were manufactured using CVI into 3D braided preforms [16]. The volume fraction of fibers was approximately 45%, and the porosity content was about 17%. The dog-bone shaped specimens were cut from composite panels using the diamond tooling, and then coated with a SiC coating. The tension-tension fatigue tests at room temperature and 1300 °C in vacuum were conducted on a servohydraulic mechanical testing machine. The fatigue experiments were performed under load control at a loading frequency of 60 Hz. The fatigue load ratio (*σ*_min_/*σ*_max_) was 0.1, and the maximum number of applied cycles was defined to be 1,000,000 cycles.

## 3. Stress Analysis

**Figure 1:**
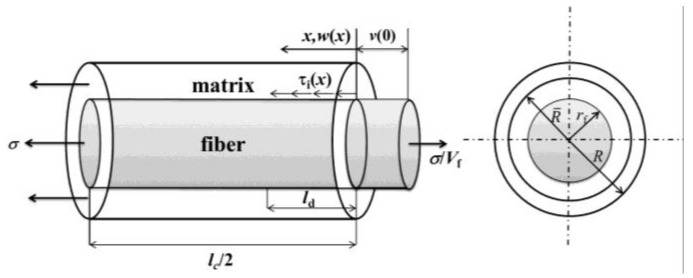
The unit cell of the Budiansky-Hutchinson-Evans shear-lag model.

To analyze stress distributions in the fiber and the matrix, a unit cell is extracted from the ceramic composite system, as shown in Figure 1. The unit cell contains a single fiber surrounded by a hollow cylinder of matrix. The fiber radius is *r*_f_ and the matrix radius is *R* (*R* = *r*_f_/*V*_f_^1/2^). The length of the unit cell is *L*/2, which is just the half matrix crack space. The interface debonded length is *L*_d_. At the matrix crack plane, fibers carry all the loads of *σ*/*V*_f_, in which *σ* denotes the far-field applied stress and *V*_f_ denotes the fiber volume content. The shear-lag model adopted by Budiansky *et al.* [17] is used to perform the stress and strain calculations in the interface debonded region (*x* ∈ [0, *L*_d_]) and interface bonded region (*x* ∈ [*L*_d_, *L*/2]).

(1) $$\sigma_{f}(x)=\left\{ \begin{array}{l} \frac{\sigma }{V_{f}}-\frac{2\tau_{i}}{r_{f}}x,x\in \left(0,L_{d}\right)\\ \sigma_{\text{fo}}+\left(\frac{V_{m}}{V_{f}}\sigma_{\text{mo}}-2\frac{L_{d}}{r_{f}}\tau_{i}\right)\exp\left(-\rho \frac{x-L_{d}}{r_{f}}\right),x\in \left(L_{d},L/2\right) \end{array}\right.$$

(2) $$\sigma_{m}(x)=\left\{ \begin{array}{l} 2\tau_{i}\frac{V_{f}}{V_{m}}\frac{x}{r_{f}},x\in \left(0,L_{d}\right)\\ \sigma_{\text{mo}}-\left(\sigma_{\text{mo}}-2\tau_{i}\frac{V_{f}}{V_{m}}\frac{L_{d}}{r_{f}}\right)\exp\left[-\frac{\rho \left(x-L_{d}\right)}{r_{f}}\right],x\in \left(L_{d},L/2\right) \end{array}\right.$$

(3) $$\tau_{i}(x)=\left\{ \begin{array}{l} \tau_{i},x\in \left(0,L_{d}\right)\\ \frac{\rho }{2}\left(\frac{V_{m}}{V_{f}}\sigma_{\text{mo}}-2\tau_{i}\frac{L_{d}}{r_{f}}\right)\exp\left[-\frac{\rho \left(x-L_{d}\right)}{r_{f}}\right],x\in \left(L_{d},L/2\right) \end{array}\right.$$

where *V*_m_ denotes the matrix volume fraction; *τ*_i_ denotes the interface shear stress; and *ρ* denotes the shear-lag model parameter [17].

(4) $$\rho^{2}=\frac{4E_{c}G_{m}}{V_{m}E_{m}E_{f}\varphi }$$

where *G*_m_ denotes the matrix shear modulus, and

(5) $$\varphi =-\frac{2\ln V_{f}+V_{m}\left(3-V_{f}\right)}{2V_{m}^{2}}$$

*σ*_fo_ and *σ*_mo_ denote the fiber and matrix axial stress in the interface bonded region, respectively.

(6) $$\sigma_{\text{fo}}=\frac{E_{f}}{E_{c}}\sigma +E_{f}\left(\alpha_{c}-\alpha_{f}\right)\Delta Τ$$

(7) $$\sigma_{\text{mo}}=\frac{E_{m}}{E_{c}}\sigma +E_{m}\left(\alpha_{c}-\alpha_{m}\right)\Delta Τ$$

where *E*_f_, *E*_m_ and *E*_c_ denote the fiber, matrix and composite elastic modulus, respectively; *α*_f_, *α*_m_ and *α*_c_ denote the fiber, matrix and composite thermal expansion coefficient, respectively; and Δ*T* denotes the temperature difference between fabricated temperature *T*_0_ and room temperature *T*_1_ (Δ*T* = *T*_1_ − *T*_0_). The axial elastic modulus of the composite is approximated by rule of mixture.

(8) $$E_{c}=V_{f}E_{f}+V_{m}E_{m}$$

When matrix multicracking and interface debonding occur, matrix cracks will serve as avenues for the ingress of the oxidizing environmental atmosphere into the composite. When the oxidizing environment ingresses into the composite, a sequence of events is triggered starting first with the oxidation of fiber coating, leading to local notch-like or neck-shrink phenomenon of fibers. As a result of this, both the axial stress distribution in the fibers and their probability of failure will change, because longer portions of the fibers are subject to peak stress *T*. During the process of oxidation, the unit cell can be divided into three regions, *i.e.*, interface oxidation region (*x* ∈ [0, *L*_t_]), interface debonded region (*x* ∈ [*L*_t_, *L*_d_]) and interface bonded region (*x* ∈ [*L*_d_, *L*/2]). When fibers fracture, the fiber axial stress distributions in the interface oxidation region, interface debonded region and interface bonded region are:

(9) $$\sigma_{f}\left(x\right)=\left\{ \begin{array}{l} T,x\in \left(0,L_{t}\right)\\ T-\frac{2\tau_{i}}{r_{f}}\left(x-L_{t}\right),x\in \left(L_{t},L_{d}\right)\\ \sigma_{\text{fo}}+\left(T-\sigma_{\text{fo}}-2\frac{L_{d}}{r_{f}}\tau_{i}\right)\exp\left(-\rho \frac{x-L_{d}}{r_{f}}\right),x\in \left(L_{d},L/2\right) \end{array}\right.$$

where *T* denotes the intact fiber axial stress at the matrix crack plane.

## 4. Damage Models

### 4.1. Matrix Multicracking

When loading fiber-reinforced CMCs, cracks typically initiate within the matrix since the strain-to-failure of matrix is usually less than that of fiber. The matrix crack spacing decreases with the increases in stress above initial matrix cracking stress *σ*_mc_ and may eventually approach saturation at stress *σ*_sat._ There are four dominant failure criterions presented in literature for modeling matrix multicrackng evolution in fiber-reinforced CMCs, *i.e.*, the maximum stress criterion, energy balance approach, critical matrix strain energy criterion and statistical failure approach. The maximum stress criterion [18] assumes that a new matrix crack will form whenever the matrix stress exceeds the ultimate strength of matrix, which is assumed to be single-valued and a known material property. The energy balance failure criteria involves calculation of the energy balance relationship before and after the formation of a single dominant crack as originally proposed by Aveston *et al.* [19]. The progression of matrix cracking as determined by energy criterion is dependent upon the matrix strain energy release rate. The energy criterion is represented by Zok and Spearing [20] and Zhu and Weitsman [21]. The concept of a critical matrix strain energy criterion [22] presupposes the existence of an ultimate or critical strain energy limit beyond which the matrix fails. Beyond this, as more energy is placed into the composite, the matrix, unable to support the additional load, continues to fail. As more energy is placed into the system, matrix fails such that all the additional energy is transferred to fibers. Failure may consist of the formation of matrix cracks, propagation of existing cracks or interface debonding. Statistical failure approach [23] assumes that matrix multicracking is governed by statistical relations, which relate the size and spatial distribution of matrix flaws to their relative propagation stress. The brittle nature of matrix material and the possible formation of initial crack distribution throughout the microstructure suggest that a statistical approach to matrix multicracking evolution is warranted in fiber-reinforced CMCs.

The tensile strength of brittle matrix is assumed to be described by two-parameter Weibull distribution where the probability of the matrix failure *P*_m_ is [23]:

(10) $$P_{m}=1-\exp\left\{-{\left[\frac{\sigma -\left(\sigma_{\text{mc}}-\sigma_{\text{th}}\right)}{\left(\sigma_{R}-\sigma_{\text{th}}\right)-\left(\sigma_{\text{mc}}-\sigma_{\text{th}}\right)}\right]}^{m}\right\}$$

where *σ*_R_ denotes the matrix characteristic strength; *σ*_mc_ denotes the matrix initial cracking stress; *σ*_th_ denotes the matrix thermal residual stress; and *m* denotes the matrix Weibull modulus.

**Figure 2:**
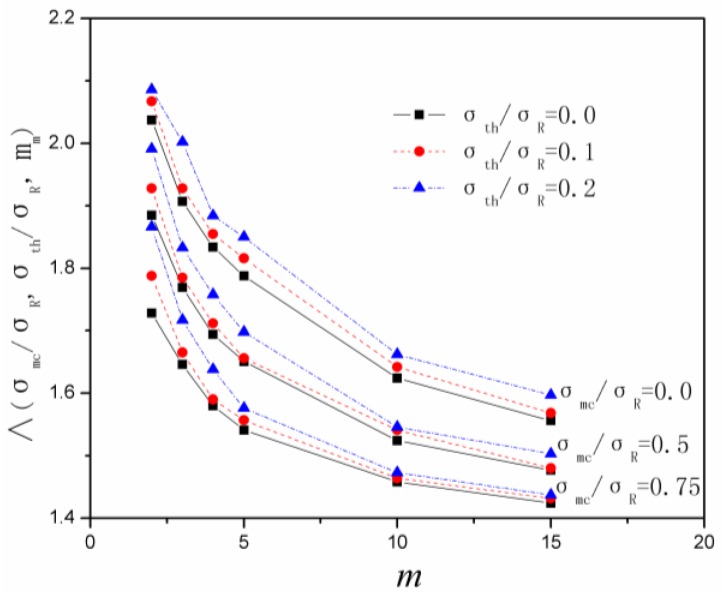
The final nominal matrix crack spacing *versus* matrix Weibull modulus of various *σ*_mc_/*σ*_R_ and *σ*_th_/*σ*_R_.

As applied stress increases, the number of matrix crack increases and matrix crack space decreases. To estimate the instantaneous matrix crack space with the increase of applied stress, it leads to the form of

(11) $$P_{m}=L_{\text{sat}}/L$$

where

(12) $$L_{\text{sat}}=\Lambda \left(\sigma_{\text{mc}}/\sigma_{R},\sigma_{\text{th}}/\sigma_{R},m\right)\delta_{R}$$

where Λ denotes the final nominal crack space, which is a pure number and depends only upon the micromechanical and statistical quantities characterizing the cracking. The final nominal crack space *versus* matrix Weibull modulus simulated by Monte Carlo method when *σ*_mc_/*σ*_R_ = 0, 0.5, 0.75 and *σ*_th_/*σ*_R_ = 0, 0.1, 0.2 are plotted in Figure 2. *δ*_R_ denotes characteristic interface sliding length.

(13) $$\delta_{R}=r_{f}\frac{V_{m}E_{m}}{V_{f}E_{c}}\frac{\sigma_{R}}{2\tau_{i}}$$

Using Equations (10) and (11), the instantaneous matrix crack space is derived by [23]

(14) $$L=r_{f}\frac{V_{m}E_{m}}{V_{f}E_{c}}\frac{\sigma_{R}}{2\tau_{i}}\Lambda {\left\{1-\exp\left[-{\left(\frac{\sigma -\left(\sigma_{\text{mc}}-\sigma_{\text{th}}\right)}{\left(\sigma_{R}-\sigma_{\text{th}}\right)-\left(\sigma_{\text{mc}}-\sigma_{\text{th}}\right)}\right)}^{m}\right]\right\}}^{-1}$$

### 4.2. Interface Debonding

When the matrix crack propagates to the fiber/matrix interface, it deflects along the interface. There are two approaches to the problem of interface debonding, *i.e.*, the shear strength approach and the fracture mechanics approach. The shear strength approach is based upon a maximum shear stress criterion in which the interface debonding occurs as the shear stress reaches the interface shear strength [24]. On the other hand, the fracture mechanics approach treats the interface debonding as a particular crack propagation problem in which the interface debonding occurs as the strain energy release rate of the interface achieves the debonded toughness [25]. It has been proved that the fracture mechanics approach is preferred to the shear strength approach for interface debonding [26]. The fracture mechanics approach is adopted in the present analysis. The interface debonding criterion is [25]

(15) $$\zeta_{d}=\frac{F}{4\pi r_{f}}\frac{\partial w_{f}\left(0\right)}{\partial L_{d}}-\frac{1}{2}\int_{0}^{L_{d}}\tau_{i}\frac{\partial v\left(x\right)}{\partial L_{d}}dx$$

where *F*(=*πr*_f_^2^*σ*/*V*_f_) denotes the fiber load at the matrix cracking plane; *w*_f_(0) denotes the fiber axial displacement on the matrix cracking plane; and *v*(*x*) denotes the relative displacement between the fiber and the matrix. The axial displacements of the fiber and the matrix, *i.e.*, *w*_f_(*x*) and *w*_m_(*x*), are

(16) $$\begin{array}{ccc} w_{f}\left(x\right) & = & \int_{x}^{L/2}\frac{\sigma_{f}}{E_{f}}dx\\ & = & \frac{T}{E_{f}}\left(L_{d}-x\right)-\frac{\tau_{i}}{r_{f}E_{f}}\left(L_{d}^{2}-x^{2}\right)+\frac{\sigma_{\text{fo}}}{E_{f}}\left(\frac{L}{2}-L_{d}\right)+\frac{r_{f}}{\rho E_{f}}\left(T-\sigma_{\text{fo}}-2\frac{L_{d}}{r_{f}}\tau_{i}\right) \end{array}$$

(17) $$\begin{array}{ccc} w_{m}\left(x\right) & = & \int_{x}^{L/2}\frac{\sigma_{m}}{E_{m}}dz\\ & = & \frac{V_{f}\tau_{i}}{r_{f}V_{m}E_{m}}\left(L_{d}^{2}-x^{2}\right)+\frac{\sigma_{\text{mo}}}{E_{m}}\left(\frac{L}{2}-L_{d}\right)-\frac{r_{f}V_{f}}{\rho V_{m}E_{m}}\left(T-\sigma_{\text{fo}}-2\tau_{i}\frac{L_{d}}{r_{f}}\right) \end{array}$$

Using Equations (16) and (17), the relative displacement between the fiber and the matrix, *i.e.*, *v*(*x*), is

(18) $$\begin{array}{ccc} v\left(x\right) & = & \left|w_{f}\left(x\right)-w_{m}\left(x\right)\right|\\ & = & \frac{T}{E_{f}}\left(L_{d}-x\right)-\frac{E_{c}\tau_{i}}{r_{f}V_{m}E_{m}E_{f}}\left(L_{d}^{2}-x^{2}\right)+\frac{r_{f}E_{c}}{\rho V_{m}E_{m}E_{f}}\left(T-\sigma_{\text{fo}}-2\frac{L_{d}}{r_{f}}\tau_{i}\right) \end{array}$$

Substituting *w*_f_(*x* = 0) and *v*(*x*) into Equation (15), it leads to the form of

(19) $$\frac{E_{c}\tau_{i}{}^{2}}{r_{f}V_{m}E_{m}E_{f}}L_{d}{}^{2}+\left(\frac{E_{c}\tau_{i}{}^{2}}{\rho V_{m}E_{m}E_{f}}-\frac{\tau_{i}T}{E_{f}}\right)L_{d}+\left(\frac{r_{f}T^{2}}{4E_{f}}-\frac{r_{f}T\sigma }{4E_{c}}-\frac{r_{f}\tau_{i}T}{2\rho E_{f}}-\zeta_{d}\right)=0$$

To solve Equation (19), the interface debonded length *L*_d_ is

(20) $$L_{d}=\frac{r_{f}}{2}\left(\frac{V_{m}E_{m}}{E_{c}\tau_{i}}T-\frac{1}{\rho }\right)-\sqrt{{\left(\frac{r_{f}}{2\rho }\right)}^{2}-\frac{r_{f}^{2}V_{f}V_{m}E_{f}E_{m}T}{4E_{c}^{2}\tau_{i}{}^{2}}\left(T-\frac{\sigma }{V_{f}}\right)+\frac{r_{f}V_{m}E_{m}E_{f}}{E_{c}\tau_{i}{}^{2}}\zeta_{d}}$$

### 4.3. Interface Wear

Upon cyclic loading of fiber-reinforced CMCs, the slip displacement between the fiber and the matrix could lead to interface wear. Evidence of interface wear that a reduction in the height of asperities occurs along the fiber coating for different thermal misfits, surface roughness and frictional sliding velocity has been presented by push-out and push-back tests on a ceramic composite system [27]. The interface wear process can be facilitated by temperature rising that occurs along the fiber/matrix interface, as frictional dissipation proceeds [28,29], *i.e.*, the temperature rising exceeded 100 K under fatigue loading at 75 Hz between stress levels of 220 and 10 MPa in unidirectional SiC/CAS-II composite [28]. Evans *et al.* [6] presented the experimental hysteresis loops data along with numerical estimates of *τ*_i_(*N*) for a unidirectional SiC/CAS composite subjected to tension-tension fatigue. The variation in interface shear stress *τ*_i_(*N*), as provided by Evans *et al.* [6] is given by Equation (21).

(21) $$\tau_{i}\left(N\right)=\tau_{\text{io}}+\left[1-\exp\left(-\omega N^{\lambda }\right)\right]\left(\tau_{\text{imin}}-\tau_{\text{io}}\right)$$

where *τ*_io_ denotes the initial interface shear stress; *τ*_imin_ denotes the steady-state interface shear stress under cyclic loading; *N* denotes the cycle number; *τ*_i_(*N*) denotes the interface shear stress at the *N*th cycle; and *ω* and *λ* are empirical constants.

Lee and Stinchcomb [30] performed the fiber fracture mirror experiments of fiber-reinforced CMCs under scanning electron microscope (SEM), and found that the fiber strength degraded with applied cycles increasing subjected to fatigue loading. The variation in fibers strength *σ*_o_(*N*), as provided by Lee and Stinchcomb [30], is given by Equation (22).

(22) $$\sigma_{o}\left(N\right)=\sigma_{o}\left[1-p_{1}{\left(\log N\right)}^{p_{2}}\right]$$

where *p*_1_ and *p*_2_ are empirical parameters.

When interface shear stress decreases with the increase of cycle number, there would be fewer loads transferred back to matrix in the interface debonded region; when the fiber strength decreases with the increase of cycle number, the load carry capability of fibers would decrease. The intact fibers are subjected to higher stress levels and the probabilities of fiber fracture would increase with the increase of cycle number.

### 4.4. Interface and Fibers Oxidation

**Figure 3:**
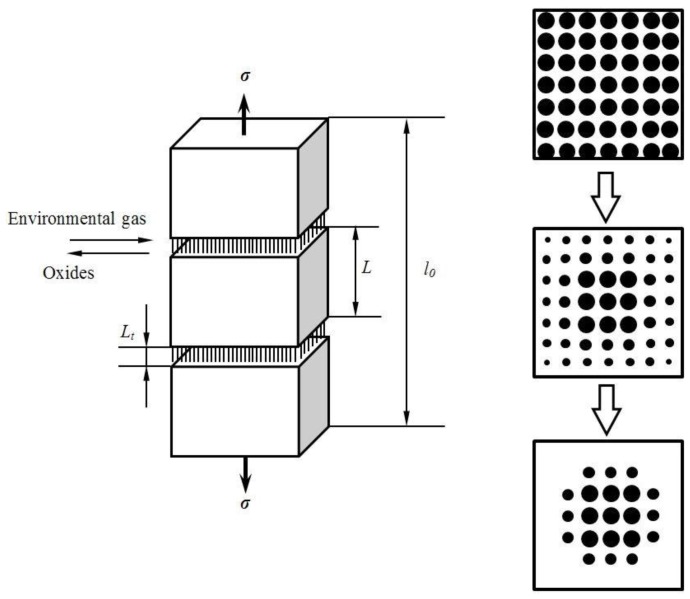
The schematic of fiber oxidation in multiple cracked C/SiC composite.

Matrix cracks will serve as avenues for the ingress of environmental atmosphere into the composite [31,32,33]. In the present study, the oxidation of fiber is assumed to be controlled by diffusion of oxygen gas through matrix cracks, as shown in Figure 3. When the oxidizing gas ingresses into the composite, a sequence of events is triggered starting first with the oxidation of the fiber. For simplicity, it is assumed that both the Weibull and elastic moduli of the fibers remain constant and that the only effect of oxidation is to decrease the strength of fibers. The time-dependent strength of fibers will be controlled by surface defects resulting from oxidation, with the thickness of the oxidized layer representing the size of the average strength-controlling flaw [34].

According to linear elastic fracture mechanics, the relationship between strength and flaw size is given by [35]

(23) $$K_{\text{IC}}=Y\sigma_{0}\sqrt{a}$$

where *K*_IC_ denotes the critical stress intensity factor; *Y* is a geometric parameter; *σ*_0_ is the fiber strength; and *a* is the size of the strength-controlling flaw.

Considering that the oxidation of fibers is controlled by diffusion of oxygen through oxidized layer, the oxidized layer will grow on the fiber’s surface according to [35].

(24) $$\alpha =\sqrt{kt}$$

where *α* is the thickness of the oxidized layer at time *t*; and *k* is the parabolic rate constant.

By assuming the fracture toughness of the fibers remains constant and that the fiber strength *σ*_0_, is related to the mean oxidized layer thickness according to Equation (22), *i.e.*, *a* = *α*, then the time dependence of the fiber strength will be given by [35].

(25) $$\sigma_{0}\left(t\right)=\sigma_{0},t\leq \frac{1}{k}{\left(\frac{K_{\text{IC}}}{Y\sigma_{0}}\right)}^{4}$$

(26) $$\sigma_{0}\left(t\right)=\frac{K_{\text{IC}}}{Y\sqrt[{4}]{kt}},t>\frac{1}{k}{\left(\frac{K_{\text{IC}}}{Y\sigma_{0}}\right)}^{4}$$

Equation (23) indicates that there exists an incubation period equal to the time required to grow an oxidized layer as thick as the size of the average critical flaw in the virgin fibers. Also note that afterwards the characteristic fiber strength changes with time as ≈*t*^−1/4^.

Filipuzzi and Naslain [33] have measured and modelled the change in the interface oxidation length *L_t_* of the carbon interface that oxidation occurs according to

(27) $$C+O_{2}\to {\text{CO}}_{2}$$

The oxidation region length of *L_t_* is [33]

(28) $$L_{t}=\varphi_{1}\left(1-e^{-\varphi_{2}t}\right)$$

where ϕ_1_ and ϕ_2_ are fitting parameters dependent on temperature. Casas *et al.* [36] performed the thermodynamic calculations and found that the deceleration of the oxidation phenomena, as a consequence of the reduced oxygen activity due to the diffusion through the glassy phases, can represent several orders of magnitude in the oxidation time scale. This effect has been incorporated into the model using a delay factor *b* in Equation (28), which becomes [36]

(29) $$L_{t}=\varphi_{1}\left(1-e^{-\frac{\varphi_{2}t}{b}}\right)$$

## 5. Fatigue Life Prediction Model

When fibers begin to break, the load dropped by broken fibers would be transferred to intact fibers in the cross-section. Two dominant failure criterions are present in the literatures for modeling fibers failure, *i.e.*, Global Load Sharing criterion (GLS) [37] and Local Load Sharing criterion (LLS) [38]. The GLS criterion assumes that the load from any one fiber is transferred equally to all other intact fibers at the same cross-section plane. The GLS assumption neglects any local stress concentrations in the neighborhood of existing breaks, and is expected to be accurate when the interface shear stress is sufficiently low. The two-parameter Weibull model is adopted to describe the fiber strength distribution. The fiber fracture probability *P*(*T*) is [39]

(30) $$P\left(T\right)\text{=1}-\text{exp}\left(-\int_{L_{0}}\frac{1}{l_{0}}{\left[\frac{\sigma_{f}\left(x\right)}{\sigma_{0}}\right]}^{m_{f}}dx\right)$$

where *σ*_0_ denotes the fiber strength at tested gauge length of *l*_0_; *m*_f_ denotes the fiber Weibull modulus; and *L*_0_ denotes the integral length.

### 5.1. Life Prediction Model at Room Temperature

The GLS assumption is used to determine the load carried by intact and fractured fibers.

(31) $$\frac{\sigma }{V_{f}}=T\left[1-P\left(T\right)\right]+\langle T_{b}\rangle P\left(T\right)$$

where <*T*_b_> denotes the average stress carried by broken fibers.

(32) $$\begin{array}{cc} \langle T_{b}\rangle = & \frac{T}{P\left(T\right)}{\left(\frac{\sigma_{c}}{T}\right)}^{m_{f}+1}{\left(\frac{\sigma_{o}\left(N\right)}{\sigma_{o}}\right)}^{m_{f}}\frac{\tau_{i}\left(N\right)}{\tau_{i}}\left\{1-\exp\left[-{\left(\frac{T}{\sigma_{c}}\right)}^{m_{f}+1}{\left(\frac{\sigma_{o}}{\sigma_{o}\left(N\right)}\right)}^{m_{f}}\frac{\tau_{i}}{\tau_{i}\left(N\right)}\right]\right\}\\ & -\frac{T}{P\left(T\right)}\exp\left\{-{\left(\frac{T}{\sigma_{c}}\right)}^{m_{f}+1}{\left(\frac{\sigma_{o}}{\sigma_{o}\left(N\right)}\right)}^{m_{f}}\frac{\tau_{i}}{\tau_{i}\left(N\right)}\right\} \end{array}$$

and

(33) $$P\left(T\right)=1-\exp\left\{-{\left(\frac{T}{\sigma_{c}}\right)}^{m_{f}+1}{\left(\frac{\sigma_{o}}{\sigma_{o}\left(N\right)}\right)}^{m_{f}}\frac{\tau_{i}}{\tau_{i}\left(N\right)}\right\}$$

Substituting Equations (32) and (33) into Equation (31), it leads to the form

(34) $$\frac{\sigma }{V_{f}}=T{\left(\frac{\sigma_{c}}{T}\right)}^{m_{f}+1}{\left(\frac{\sigma_{o}\left(N\right)}{\sigma_{o}}\right)}^{m_{f}}\frac{\tau_{i}\left(N\right)}{\tau_{i}}\left\{1-\exp\left[-{\left(\frac{T}{\sigma_{c}}\right)}^{m_{f}+1}{\left(\frac{\sigma_{o}}{\sigma_{o}\left(N\right)}\right)}^{m_{f}}\frac{\tau_{i}}{\tau_{i}\left(N\right)}\right]\right\}$$

Using Equations (21), (22) and (34), the stress *T* carried by intact fibers at the matrix cracking plane can be determined for different fatigue peak stresses. Substituting Equations (21) and (22) and the intact fibers stress *T* into Equation (33), the fiber failure probability corresponding to different cycle number can be determined. When the broken fiber fraction approaches the critical value, the composites fatigue fracture.

### 5.2. Life Prediction at Elevated Temperatures in the Oxidative Environment

When fiber-reinforced CMCs aree subjected to oxidation, a notch would form at the fiber surface leading to the degradation of fiber strength and the increase of fiber stress concentration and fracture probability. The fracture probabilities of oxidized fibers in the oxidation region, unoxidized fibers in the oxidation region, fibers in the interface debonded region and interface bonded region of *P_a_*(*T*), *P_b_*(*T*), *P_c_*(*T*) and *P_d_*(*T*) are

(35) $$P_{a}\left(T\right)=1-\text{exp}\left\{-2\frac{L_{t}}{l_{0}}{\left[\frac{T}{\sigma_{0}\left(t\right)}\right]}^{m_{f}}\right\}$$

(36) $$P_{b}\left(T\right)=1-\text{exp}\left\{-2\frac{L_{t}}{l_{0}}{\left(\frac{T}{\sigma_{0}}\right)}^{m_{f}}\right\}$$

(37) $$P_{c}\left(T\right)=1-\exp\left\{-\frac{r_{f}T^{m_{f}+1}}{l_{0}{\left(\sigma_{0}\left(N\right)\right)}^{m_{f}}\tau_{i}\left(N\right)\left(m_{f}+1\right)}\left[1-{\left(1-\frac{L_{d}\left(N\right)}{l_{f}\left(N\right)}\right)}^{m_{f}+1}\right]\right\}$$

(38) $$\begin{array}{l} P_{d}\left(T\right)=1-\exp\{-\frac{2r_{f}T^{m_{f}}}{\rho l_{0}{\left(\sigma_{0}\left(N\right)\right)}^{m_{f}}\left(m_{f}+1\right)\left(1-\frac{\sigma_{\text{fo}}}{T}-\frac{L_{d}\left(N\right)}{l_{s}\left(N\right)}\right)}\times [(1-\frac{L_{d}\left(N\right)}{l_{f}\left(N\right)}-\\ {\left(1-\frac{\sigma_{\text{fo}}}{T}-\frac{L_{d}\left(N\right)}{l_{f}\left(N\right)}\right)\frac{\rho L_{d}\left(N\right)}{r_{f}})}^{m_{f}+1}-{\left(1-\frac{L_{d}\left(N\right)}{l_{f}\left(N\right)}-\left(1-\frac{\sigma_{\text{fo}}}{T}-\frac{L_{d}\left(N\right)}{l_{f}\left(N\right)}\right)\frac{\rho L}{2r_{f}}\right)}^{m_{f}+1}]\} \end{array}$$

where *l*_f_ denotes the slip length over which the fiber stress would decay to zero if not interrupted by the far-field equilibrium stresses.

(39) $$l_{f}\left(N\right)=\frac{r_{f}T}{2\tau_{i}\left(N\right)}$$

The GLS assumption is used to determine the load carried by intact and fracture fibers [39].

(40) $$\frac{\sigma }{V_{f}}=\left[1-P_{f}\left(T\right)\left(1+\frac{2l_{f}}{L}\right)\right]T+P_{r}\left(T\right)\frac{2l_{f}}{L}\langle T_{b}\rangle$$

where

(41) $$P_{f}\left(T\right)=\varphi \left[\eta P_{a}\left(T\right)+\left(1-\eta \right)P_{b}\left(T\right)\right]+P_{c}\left(T\right)+P_{d}\left(T\right)$$

(42) $$P_{r}\left(T\right)=P_{c}\left(T\right)+P_{d}\left(T\right)$$

where *η* denotes the oxidation fibers fraction in the oxidized region; and φ denotes the fraction of oxidation in the multiple matrix cracks.

(43) $$\varphi =\frac{L_{\text{sat}}}{l_{f}-2L_{t}}$$

The average stress carried by broken fibers is given by Equation (44).

(44) $$\begin{array}{l} \langle T_{b}\rangle =\int_{0}^{l_{f}}T_{b}\left(x\right)f\left(x\right)dx\\ =\frac{T}{P_{r}\left(T\right)}{\left(\frac{\sigma_{c}}{T}\right)}^{m_{f}+1}{\left(\frac{\sigma_{o}\left(N\right)}{\sigma_{o}}\right)}^{m_{f}}\frac{\tau_{i}\left(N\right)}{\tau_{i}}\left\{1-\exp\left[-{\left(\frac{T}{\sigma_{c}}\right)}^{m_{f}+1}{\left(\frac{\sigma_{o}}{\sigma_{o}\left(N\right)}\right)}^{m_{f}}\frac{\tau_{i}}{\tau_{i}\left(N\right)}\right]\right\}\\ -\frac{T}{P_{r}\left(T\right)}\exp\left\{-{\left(\frac{T}{\sigma_{c}}\right)}^{m_{f}+1}{\left(\frac{\sigma_{o}}{\sigma_{o}\left(N\right)}\right)}^{m_{f}}\frac{\tau_{i}}{\tau_{i}\left(N\right)}\right\} \end{array}$$

Substituting Equations (41), (42) and (44) into Equations (35)–(38), the stress *T* carried by intact fibers at the matrix crack plane can be determined for different cycle number and fatigue stress. Substituting Equations (21), (22), (25), (26) and (29) and the intact fiber stress *T* into Equations (41) and (42), the fiber failure probabilities corresponding to different numbers of applied cycles can be determined. When the broken fiber fraction approaches the critical value, the composites fatigue fracture.

## 6. Experimental Comparisons

Under cyclic fatigue loading, the loading directions were along with fiber for the unidirectional CMCs, 0° fiber ply for the cross-ply and plain-weave 2D CMCs, warp yarn for the 2.5D CMCs, and axial fibers at a small angle *θ* for 3D CMCs. An effective coefficient of the fiber volume content along the loading direction (ECFL) is defined as:

(45) $$\psi =\frac{V_{f\_\mathrm{axial}}}{V_{f}}$$

where *V*_f_ and *V*_f_axial_ denote the total fiber volume fraction in the composites and the effective fiber volume fraction in the cyclic loading direction. Under cyclic fatigue loading at room and elevated temperatures, the broken fiber fraction in the 0° plies or longitudinal yarns of cross-ply and 2.5D CMCs would increase with the increase of loading cycles and oxidation time. When the broken fiber fraction in the 0° plies or longitudinal yarns approaches the critical value, the composite would fatigue fail.

### 6.1. Life Prediction at Room Temperature

The monotonic tensile strength of unidirectional C/SiC composite is 270 MPa, and the fatigue peak stresses are 0.51, 0.66, 0.74, 0.88 and 0.96 of tensile strength; the monotonic tensile strength of cross-ply C/SiC composite is 124 MPa, and the fatigue peak stresses are 0.70, 0.80, 0.85 and 0.90 of tensile strength; the monotonic tensile strength of 2D C/SiC composite is 420 MPa, and the fatigue peak stresses are 0.80, 0.83, 0.86, 0.89, 0.91 and 0.96 of tensile strength [11]; the monotonic tensile strength of 2.5D C/SiC composite is 225 MPa, and the fatigue peak stresses are 0.6, 0.7, 0.75 and 0.8 of tensile strength [14]; and the monotonic tensile strength of 3D C/SiC is 276 MPa, and the fatigue peak stresses are 0.80, 0.83, 0.87, 0.89, 0.90 and 0.94 of tensile strength [16].

**Figure 4:**
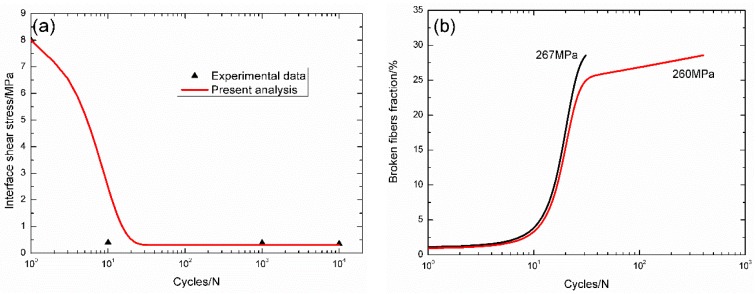

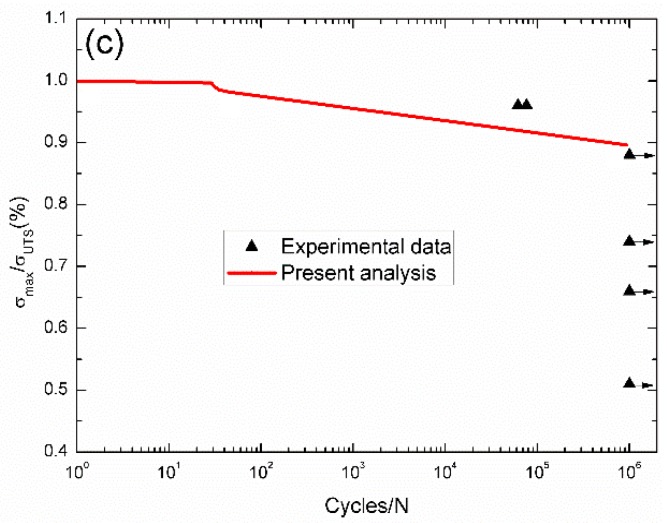
(**a**) The interface shear stress *versus* applied cycles; (**b**) the broken fibers fraction *versus* applied cycles; and (**c**) the fatigue life S–N curves of experimental data and theoretical analysis for unidirectional C/SiC composite at room temperature.

For unidirectional C/SiC composite, the interface shear stress *versus* applied cycles curve has been simulated by the Evans-Zok-McMeeking model [6], as shown in Figure 4a. The material properties are listed in Table 1. The broken fiber fraction *versus* cycle number curves under *σ*_max_ = 267 and 260 MPa are illustrated in Figure 4b. Under *σ*_max_ = 267 MPa, the composite fatigue failed after 31 cycles; and under *σ*_max_ = 260 MPa, the composite fatigue failed after 400 cycles. The experimental and theoretical fatigue life S–N curves are illustrated in Figure 4c, in which the fatigue limit approaches 88% of tensile strength.

**Figure 5:**
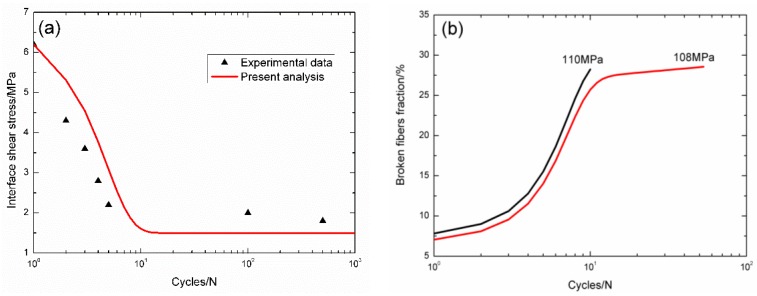

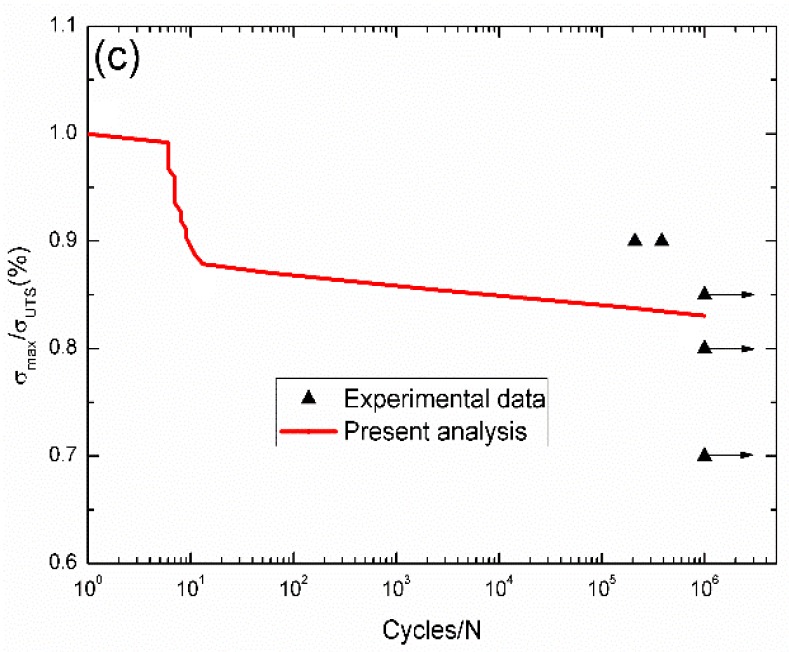
(**a**) The interface shear stress *versus* applied cycles; (**b**) the broken fibers fraction *versus* applied cycles; and (**c**) the fatigue life S–N curves of experimental data and theoretical analysis for cross-ply C/SiC composite at room temperature.

For cross-ply C/SiC composite, the interface shear stress *versus* applied cycles curve has been simulated by the Evans-Zok-McMeeking model [6], as shown in Figure 5a. The material properties are listed in Table 1. The broken fibers fraction *versus* cycle number curves under *σ*_max_ = 110 and 108 MPa are illustrated in Figure 5b. Under *σ*_max_ = 110 MPa, the composite fatigue failed after 10 cycles; and under *σ*_max_ = 108 MPa, the composite fatigue failed after 53 cycles. The experimental and theoretical fatigue life S–N curves are illustrated in Figure 5c, in which the fatigue limit approaches 88% of tensile strength.

**Figure 6:**
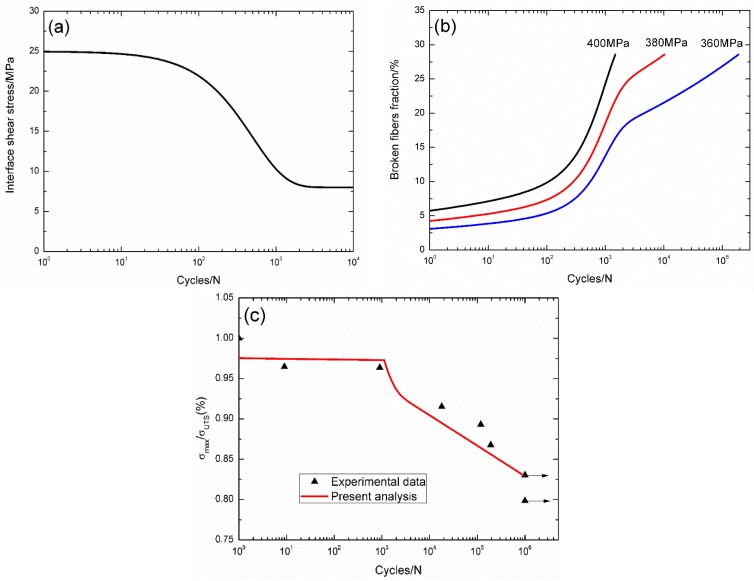
(**a**) The interface shear stress *versus* applied cycles; (**b**) the broken fibers fraction *versus* applied cycles; and (**c**) the fatigue life S–N curves of experimental data and theoretical analysis for 2D C/SiC composite at room temperature.

For 2D C/SiC composite [11], the interface shear stress *versus* applied cycles curve has been simulated using the Evans-Zok-McMeeking model [6], as shown in Figure 6a. The material properties are listed in Table 1. The broken fibers fraction *versus* cycle number curves under *σ*_max_ = 400, 380 and 360 MPa are illustrated in Figure 6b. Under *σ*_max_ = 400 MPa, the composite fatigue failed after 1487 cycles; under *σ*_max_ = 380 and 360 MPa, the composite failed after 10,312 and 189,202 cycles, respectively. The experimental and theoretical fatigue life S–N curves are illustrated in Figure 6c, in which the fatigue limit approaches 85% of tensile strength.

**Figure 7:**
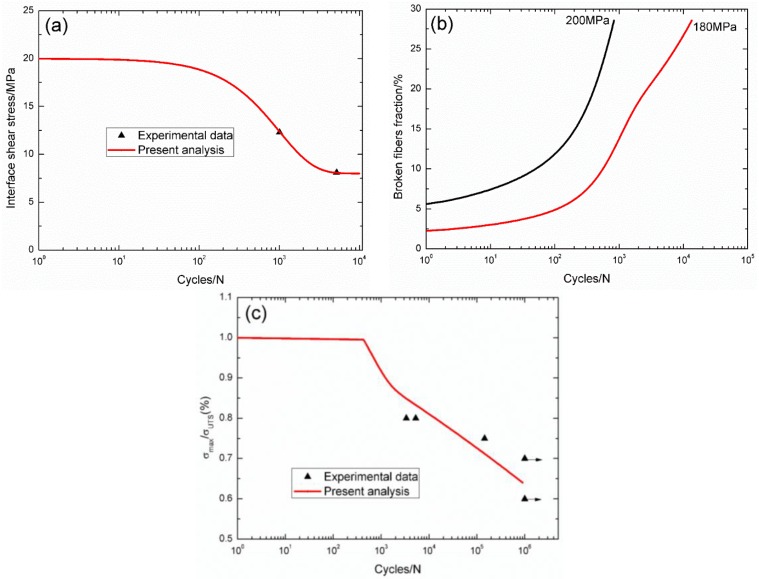
(**a**) The interface shear stress *versus* applied cycles; (**b**) the broken fibers fraction *versus* applied cycles; and (**c**) the fatigue life S–N curves of experimental data and theoretical analysis for 2.5D C/SiC composite at room temperature.

For 2.5D C/SiC composite [14], the interface shear stress *versus* applied cycles curve has been simulated by the Evans-Zok-McMeeking model [6], as shown in Figure 7a. The material properties are listed in Table 1. The broken fibers fraction *versus* cycle number curves under *σ*_max_ = 200 and 180 MPa are illustrated in Figure 7b. Under *σ*_max_ = 200 MPa, the composite fatigue failed after 832 cycles; and under *σ*_max_ = 180 MPa, the composite fatigue failed after 13,470 cycles. The experimental and theoretical fatigue life S–N curves are illustrated in Figure 7c, in which the fatigue limit approaches 70% of tensile strength.

**Figure 8:**
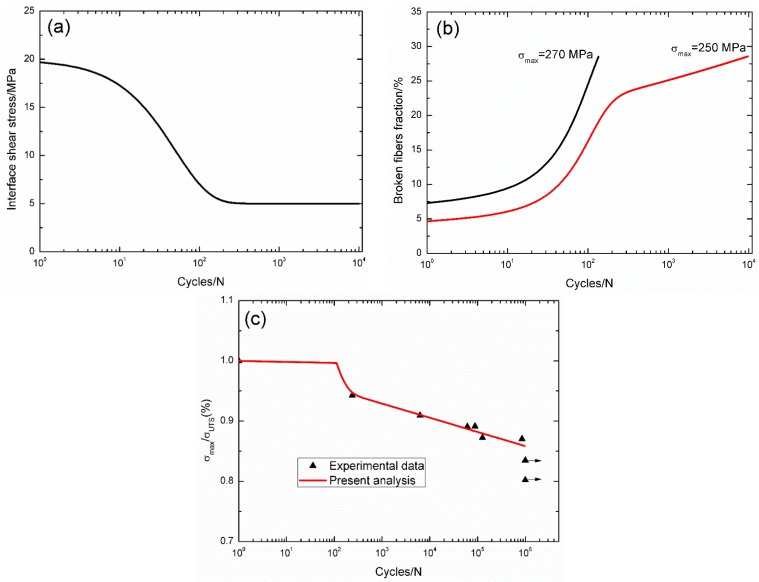
(**a**) The interface shear stress *versus* applied cycles; (**b**) the broken fibers fraction *versus* applied cycles; and (**c**) the fatigue life S–N curves of experimental data and theoretical analysis for 3D C/SiC composite at room temperature.

For the 3D C/SiC composite [16], the interface shear stress *versus* applied cycles curve has been simulated by Evans-Zok-McMeeking model [6], as shown in Figure 8a. The material properties are listed in Table 1. The broken fibers fraction *versus* cycle number curves under *σ*_max_ = 270 and 250 MPa are illustrated in Figure 8b. Under *σ*_max_ = 270 MPa, the composite fatigue failed after 135 cycles; and under *σ*_max_ = 250 MPa, the composite fatigue failed after 9754 cycles. The experimental and theoretical fatigue life S–N curves are illustrated in Figure 8c, in which the fatigue limit approaches 85% of tensile strength.

### 6.2. Life Prediction at Elevated Temperatures

The monotonic tensile strength of unidirectional C/SiC composite is 320 MPa at 800 °C in air, and the fatigue peak stresses are 0.37, 0.43, 0.56, 0.65 and 0.78 of tensile strength; the monotonic tensile strength of cross-ply C/SiC composite is 150 MPa at 800 °C in air, and the fatigue peak stresses are 0.60, 0.65 and 0.70 of tensile strength; the monotonic tensile strength of 2D C/SiC composite is 487 MPa at 550 °C in air, and the fatigue peak stresses are 0.22, 0.36, 0.56 and 0.72 of tensile strength [12]; the monotonic tensile strength of 2D C/SiC composite is 300 MPa at 1300 °C in the oxidative environment, and the fatigue peak stresses are 0.5, 0.6, 0.7 and 0.8 of tensile strength [13]; the monotonic tensile strength of 2.5D C/SiC composite is 280 MPa at 800 °C in air, and the fatigue peak stresses are 0.5, 0.6, 0.7 and 0.8 of tensile strength [14]; the monotonic tensile strength of 2.5D C/SiC composite is 228 MPa at 900 °C in air, and the fatigue peak stresses are 0.35, 0.4, 0.43, 0.52, 0.6 and 0.7 of tensile strength [15] and the monotonic tensile strength of 3D C/SiC composite is 304 MPa at 1300 °C in vacuum, and the fatigue peak stresses are 0.83, 0.5, 0.93, 0.98 and 0.99 of tensile strength [16].

**Figure 9:**
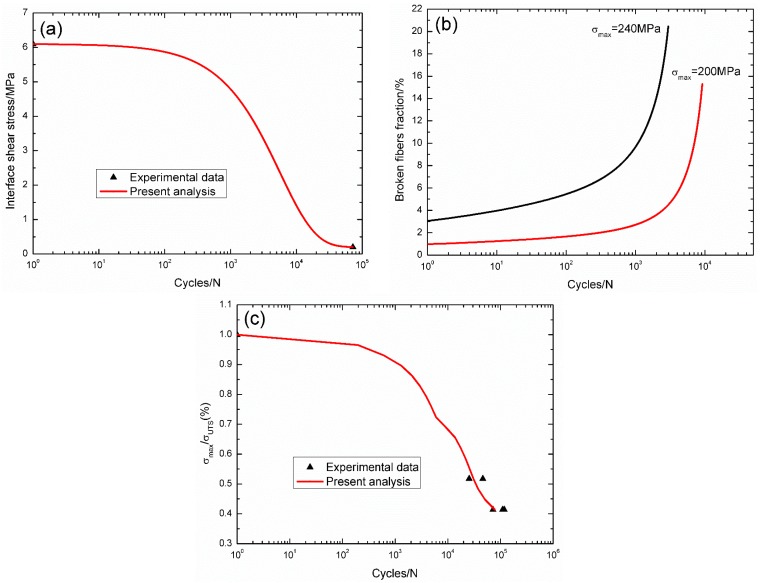
(**a**) The interface shear stress *versus* applied cycles; (**b**) the broken fibers fraction *versus* applied cycles; and (**c**) the fatigue life S–N curves of experimental data and theoretical analysis for unidirectional C/SiC composite at 800 °C in air.

For unidirectional C/SiC composite at 800 °C in air, the interface shear stress *versus* applied cycles curve has been simulated by the Evans-Zok-McMeeking model [6], as shown in Figure 9a. The material properties are listed in Table 2. The broken fibers fraction *versus* cycle number curves under *σ*_max_ = 240 and 200 MPa are illustrated in Figure 9b. Under *σ*_max_ = 240 MPa, the composite fatigue failed after 2970 cycles with the broken fibers fraction of 20.4%; and under *σ*_max_ = 200 MPa, the composite fatigue failed after 9195 cycles with the broken fiber fraction of 15.3%. The experimental and theoretical fatigue life S–N curves are given in Figure 9c, in which the fatigue life at 800 °C in air is greatly reduced compared with that at room temperature, mainly attributed to oxidation of PyC interphase and carbon fibers.

**Figure 10:**
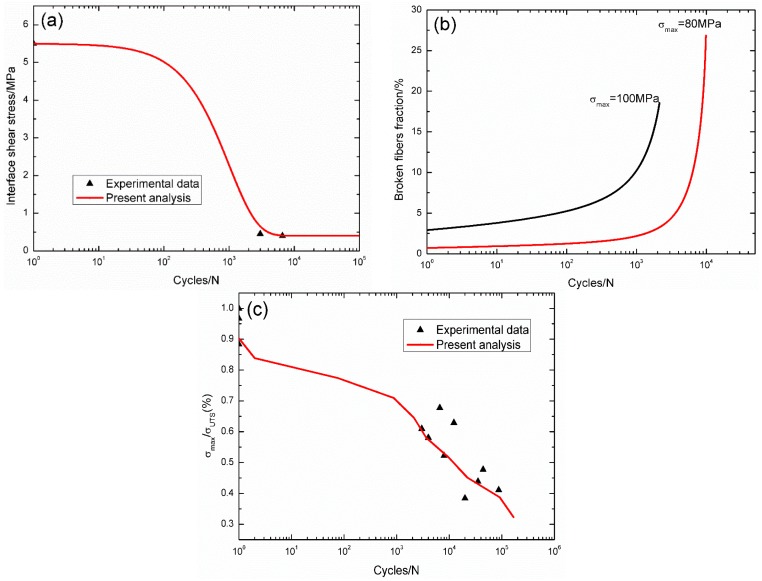
(**a**) The interface shear stress *versus* applied cycles; (**b**) the broken fiber fraction *versus* applied cycles; and (**c**) the fatigue life S–N curves of experimental data and theoretical analysis for cross-ply C/SiC composite at 800 °C in air.

For cross-ply C/SiC composite at 800 °C in air, the interface shear stress *versus* applied cycles curve has been simulated by the Evans-Zok-McMeeking model [6], as shown in Figure 10a. The material properties are listed in Table 2. The broken fibers fraction *versus* cycle number curves under *σ*_max_ = 100 and 80 MPa are illustrated in Figure 10b. Under *σ*_max_ = 100 MPa, the composite fatigue failed after 2134 cycles with the broken fibers fraction of 18.5%; and under *σ*_max_ = 80 MPa, the composite fatigue failed after 9881 cycles with the broken fibers fraction of 26.8%. The experimental and theoretical fatigue life S–N curves are illustrated in Figure 10c, in which the fatigue life at 800 °C in air is greatly reduced compared with that at room temperature, attributed to oxidation of PyC interphase and carbon fibers.

**Figure 11:**
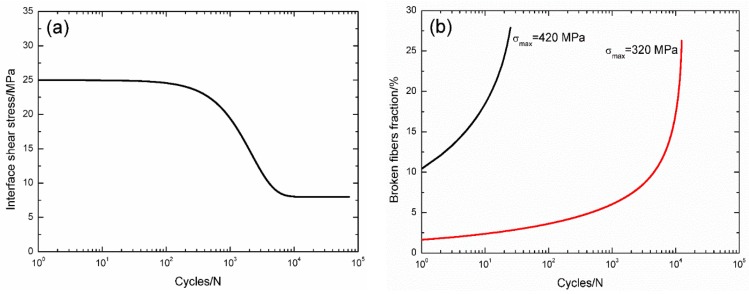

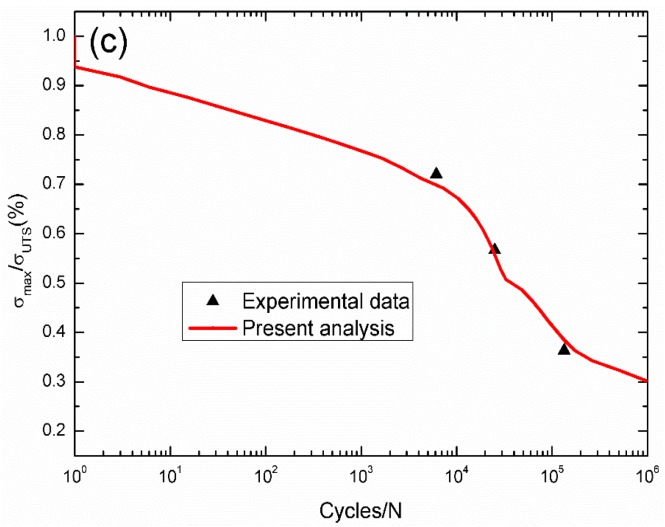
(**a**) The interface shear stress *versus* applied cycles; (**b**) the broken fiber fraction *versus* applied cycles; and (**c**) the fatigue life S–N curves of experimental data and theoretical analysis for 2D woven C/SiC composite at 550 °C in air.

For 2D C/SiC composite at 550 °C in air [12], the interface shear stress *versus* applied cycles has been simulated by the Evans-Zok-McMeeking model [6], as shown in Figure 11a. The material properties are listed in Table 2. The broken fiber fraction *versus* cycle number curves under *σ*_max_ = 420 and 320 MPa are illustrated in Figure 11b. Under *σ*_max_ = 420 MPa, the composite fatigue failed after 25 cycles with the broken fiber fraction of 27.8%; and under *σ*_max_ = 320 MPa, the composite fatigue failed after 12,457 cycles with the broken fiber fraction of 26.2%. The experimental and theoretical fatigue life S–N curves are illustrated in Figure 11c, in which the fatigue life at 550 °C in air is greatly reduced compared with that at room temperature, mainly attributed to oxidation of interphase and carbon fibers.

**Figure 12:**
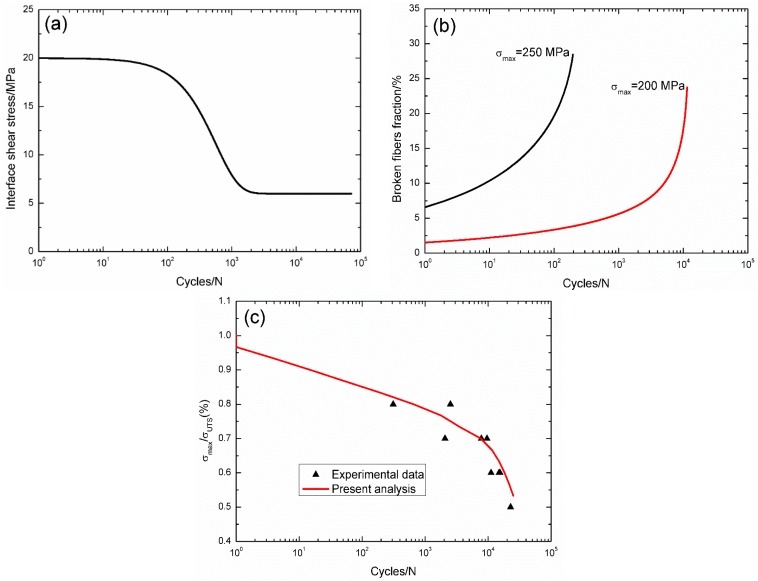
(**a**) The interface shear stress *versus* applied cycles; (**b**) the broken fiber fraction *versus* applied cycles; and (**c**) the fatigue life S–N curves of experimental data and theoretical analysis for 2D C/SiC composite at 1300 °C in the oxidative atmosphere.

For 2D C/SiC composite at 1300 °C in the oxidative environment [13], the interface shear stress *versus* applied cycles has been simulated by the Evans-Zok-McMeeking model [6], as shown in Figure 12a. The material properties are listed in Table 2. The broken fiber fraction *versus* cycle number curves under *σ*_max_ = 250 and 200 MPa are illustrated in Figure 12b. Under *σ*_max_ = 250 MPa, the composite fatigue failed after 196 cycles with the broken fiber fraction of 28.4%; and under *σ*_max_ = 200 MPa, the composite fatigue failed after 11,480 cycles with the broken fiber fraction of 23.7%. The experimental and theoretical fatigue life S–N curves are illustrated in Figure 12c, in which the fatigue life at 1300 °C in the oxidative environment is greatly reduced compared with that at room temperature, mainly attributed to oxidation of interphase and carbon fibers.

**Figure 13:**
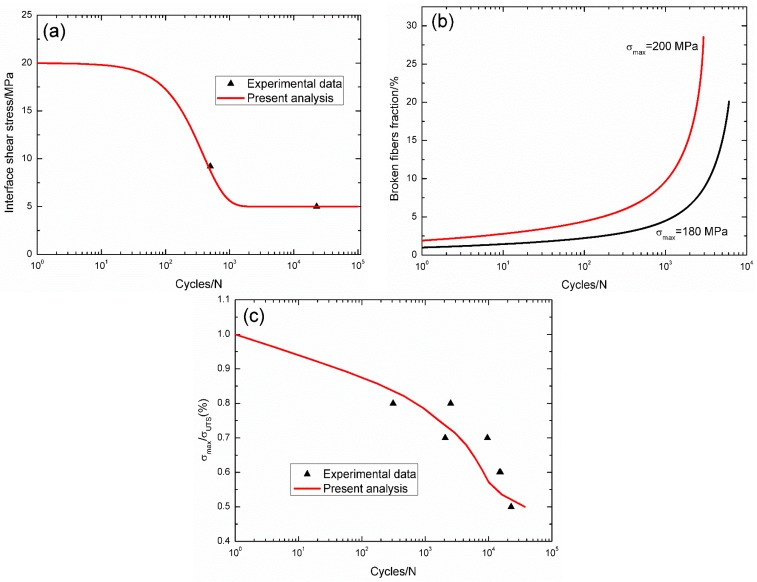
(**a**) The interface shear stress *versus* applied cycles; (**b**) the broken fiber fraction *versus* applied cycles; and (**c**) the fatigue life S–N curves of experimental data and theoretical analysis for 2.5D C/SiC composite at 800 °C in air.

For 2.5D C/SiC composite at 800 °C in air [14], the interface shear stress *versus* applied cycles has been simulated by the Evans-Zok-McMeeking model [6], as shown in Figure 13a. The material properties are listed in Table 2. The broken fiber fraction *versus* cycle number curves under *σ*_max_ = 200 and 180 MPa are illustrated in Figure 13b. Under *σ*_max_ = 200 MPa, the composite fatigue failed after 2945 cycles with the broken fibers fraction of 28.5%; and under *σ*_max_ = 180 MPa, the composite fatigue failed after 6078 cycles with the broken fiber fraction of 20.1%. The experimental and predicted fatigue life S–N curves are illustrated in Figure 13c, in which the fatigue life at 800 °C in air is greatly reduced compared with that at room temperature, mainly attributed to oxidation of PyC interphase and carbon fibers.

**Figure 14:**
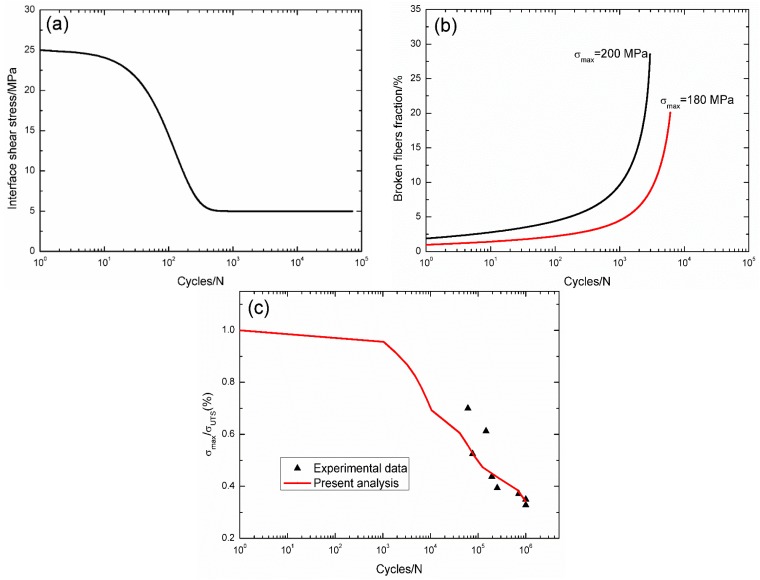
(**a**) The interface shear stress *versus* applied cycles; (**b**) the broken fiber fraction *versus* applied cycles; and (**c**) the fatigue life S–N curves of experimental data and theoretical analysis for 2.5D C/SiC composite at 900 °C in air.

For 2.5D C/SiC composite at 900 °C in air [15], the interface shear stress *versus* applied cycles has been simulated by the Evans-Zok-McMeeking model [6], as shown in Figure 14a. The material properties are listed in Table 2. The broken fiber fraction *versus* cycle number curves under *σ*_max_ = 220 and 210 MPa are illustrated in Figure 14b. Under *σ*_max_ = 220 MPa, the composite fatigue failed after 2945 cycles with the broken fibers fraction of 26.4%; and under *σ*_max_ = 210 MPa, the composite fatigue failed after 1649 cycles with the broken fiber fraction of 24.1%. The experimental and theoretical fatigue life S–N curves are illustrated in Figure 14c, in which the fatigue life at 900 °C in air is greatly reduced compared with that at room temperature, mainly attributed to oxidation of PyC interphase and carbon fibers.

**Figure 15:**
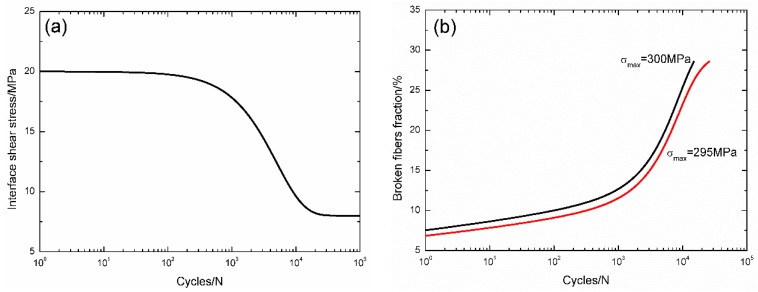

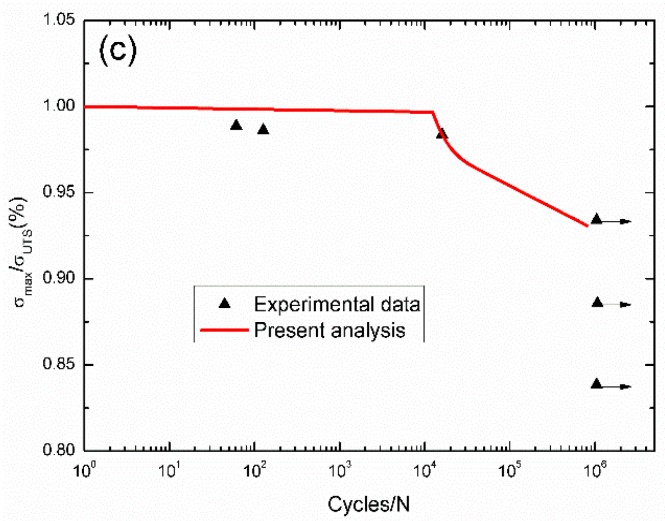
(**a**) The interface shear stress *versus* applied cycles; (**b**) the broken fiber fraction *versus* applied cycles; and (**c**) the fatigue life S–N curves of experimental data and theoretical analysis for 3D C/SiC composite at 1300 °C in vacuum.

For 3D C/SiC composite at 1300 °C in vacuum [16], the interface shear stress *versus* applied cycles has been simulated by the Evans-Zok-McMeeking model [6], as shown in Figure 15a. The material properties are listed in Table 2. The broken fiber fraction *versus* cycle number curves under *σ*_max_ = 300 and 295 MPa are illustrated in Figure 15b. Under *σ*_max_ = 300 MPa, the composite fatigue failed after 14,968 cycles with the broken fiber fraction of 28.5%; and under *σ*_max_ = 295 MPa, the composite fatigue failed after 25,773 cycles with the broken fiber fraction of 28.5%. The experimental and predicted fatigue life S–N curves at 1300 °C in vacuum are illustrated in Figure 15c, in which the fatigue life and fatigue limit at 1300 °C in vacuum are increased compared with those at room temperature.

## 7. Conclusions

An approach to predict the fatigue life of fiber-reinforced CMCs with different fiber preforms, *i.e.*, unidirectional, cross-ply, 2D, 2.5D and 3D CMCs at room and elevated temperatures in air and oxidative environments, has been developed considering the fatigue damage mechanism of interface wear at room temperature, interface and fiber oxidation at elevated temperatures. An effective coefficient of the fiber volume fraction along the loading direction (ECFL) was introduced to describe the fiber architecture of the preforms. The two-parameter Weibull model was used to describe the fibers’ strength distribution. The stress carried by broken and intact fibers on the matrix crack plane under cyclic fatigue loading was determined based on the assumption of Global Load Sharing (GLS) criterion. The broken fiber fraction under cyclic fatigue loading considering the degradation of interface shear stress and fiber strength was obtained. The fatigue life S–N curves and fatigue limit of unidirectional C/SiC, cross-ply C/SiC, 2D C/SiC, 2.5D C/SiC, and 3D C/SiC composites have been predicted.

1. The broken fiber fraction *versus* applied cycles curve can be divided into two regions, *i.e.*, at the initial loading cycles, the broken fiber fraction increases rapidly due to the degradation of interface shear stress and fiber strength; and when interface shear stress approaches the steady-state value, fibers’ failure is mainly controlled by fiber strength degradation, which makes the broken fiber fraction increase slowly.
2. The predicted fatigue life S–N curves can also be divided into two regions, *i.e.*, the region I is controlled by the degradation of interface shear stress and fiber strength; and the region II is only controlled by the degradation of fiber strength.
3. The fatigue life of unidirectional, cross-ply, 2D and 2.5D C/SiC composites at elevated temperatures in air or oxidative environments is greatly reduced compared with that at room temperature, mainly attributed to oxidation of PyC interphase and carbon fibers; however, at 1300 °C in vacuum, the fatigue life and fatigue limit increase compared with that at room temperature.

## Figures and Tables

**Table 1:** The material properties of unidirectional (UD), cross-ply (CP), 2D, 2.5D and 3D C/SiC composites at room temperature.

Items	UD [9]	CP [9]	2D [11]	2.5D [14]	3D [16]
*V*_f_/(%)	40	40	45	45	45
*ψ*	1.0	0.5	0.5	0.75	0.93
*r*_f_/(μm)	3.5	3.5	3.5	3.5	3.5
*m*_f_	5	5	5	5	5
*τ_i_*_o_/(MPa)	8	6.2	25	20	20
*τ_i_*_min_/(MPa)	0.3	1.5	8	8	5
*ω*	0.04	0.06	0.002	0.001	0.02
*λ*	1.5	1.8	1.0	1.0	1.0
*p*_1_	0.02	0.01	0.018	0.02	0.012
*p*_2_	1.0	0.8	1.0	1.2	1.0

**Table 2:** The material properties of unidirectional (UD), cross-ply (CP), 2D, 2.5D and 3D C/SiC composites at elevated temperature.

Items	UD [9]	CP [9]	2D [12]	2D [13]	2.5D [14]	2.5D [15]	3D [16]
*V*_f_/(%)	40	40	45	40	45	40	45
*ψ*	1.0	0.5	0.5	0.5	0.75	0.75	0.93
*r*_f_/(μm)	3.5	3.5	3.5	3.5	3.5	3.5	3.5
*m*_f_	5	5	5	5	5	5	5
*τ_i_*_o_/(MPa)	6.1	5.5	25	20	20	25	20
*τ_i_*_min_/(MPa)	0.2	0.4	8	6	5	5	8
*ω*	0.001	0.001	0.0001	0.0005	0.008	0.003	0.002
*λ*	0.8	1.0	1.2	1.2	1.2	1.2	1.0
*p*_1_	0.02	0.02	0.02	0.02	0.03	0.03	0.018
*p*_2_	1.0	1.0	1.0	1.0	1.2	1.2	1.0

## References

[1] Naslain R. (2004). Design, preparation and properties of non-oxide CMCs for application in engines and nuclear reactors: An overview. Compos. Sci. Technol..

[2] Bednarcyk B.A., Mital S.K., Pineda E.J., Arnold S.M. Multiscale modeling of ceramic matrix composites. Proceedings of the 56th AIAA/ASCE/AHS/ASC Structures, Structural Dynamics, and Materials Conference.

[3] Li L.B. (2016). Fatigue hysteresis of carbon fiber-reinforced ceramic-matrix composites at room and elevated temperatures. Appl. Compos. Mater..

[4] Li L.B. (2014). Modeling fatigue hysteresis behavior of unidirectional C/SiC ceramic-matrix composite. Compos. Part B.

[5] Gowayed Y., Ojard G., Santhosh U., Jefferso G. (2015). Modeling of crack density in ceramic matrix composites. J. Compos. Mater..

[6] Evans A.G., Zok F.W., McMeeking R.M. (1995). Fatigue of ceramic matrix composites. Acta Metall. Mater..

[7] Pailler F., Lamon J. (2005). Micromechanics based model of fatigue/oxidation for ceramic matrix composites. Compos. Sci. Technol..

[8] Reynaud P. (1996). Cyclic fatigue of ceramic-matrix composites at ambient and elevated temperatures. Compos. Sci. Technol..

[9] Li L.B. (2010). Fatigue damage models and life prediction of long-fiber-reinforced ceramic matrix composites. Ph.D. Thesis.

[10] American Society for Testing Materials (2015). Standard Practice for Constant-Amplitude, Axial, Tension-Tension Cyclic Fatigue of Continuous Fiber-Reinforced Advanced Ceramics at Ambient Temperatures.

[11] Shuler S.F., Holmes J.W., Wu X. (1993). Influence of loading frequency on the room-temperature fatigue of a carbon-fiber/SiC-matrix composite. J. Am. Ceram. Soc..

[12] Mall S., Engesser J.M. (2006). Effects of frequency on fatigue behavior of CVI C/SiC at elevated temperature. Compos. Sci. Technol..

[13] Cheng Q.Y., Tong X.Y., Zheng X., Zhou J., Yao L.J., Li B. (2010). Experimental investigation on the fatigue characteristics about high temperature of plain-woven C/SiC composite. J. Mech. Strength.

[14] Yang F.S. (2011). Research on Fatigue Behavior of 2.5D Woven Ceramic Matrix Composites. Master’s Thesis.

[15] Zhang C.Y., Wang X.W., Liu Y.S., Wang B., Han D., Qiao S., Guo Y. (2013). Tensile fatigue of a 2.5D-C/SiC composite at room temperature and 900 °C. Mater. Des..

[16] Du S.M., Qiao S.R., Ji G.C., Han D. (2002). Tension-tension fatigue behavior of 3D-C/SiC composite at room temperature and 1300 °C. Mater. Eng..

[17] Budiansky B., Hutchinson J.W., Evans A.G. (1986). Matrix fracture in fiber-reinforced ceramics. J. Mech. Phys. Solids.

[18] Daniel I.M., Lee J.W. (1993). The behavior of ceramic matrix fiber composites under longitudinal loading. Compos. Sci. Technol..

[19] Aveston J., Cooper G.A., Kelly A. (1971). Single and multiple fracture. The Properties of Fiber Composites.

[20] Zok F.W., Spearing S.M. (1992). Matrix crack spacing in brittle matrix composites. Acta Metall. Mater..

[21] Zhu H., Weitsman Y. (1994). The progression of failure mechanisms in unidirectional reinforced ceramic composites. J. Mech. Phys. Solids.

[22] Solti J.P., Mall S., Robertson D.D. (1995). Modeling damage in unidirectional ceramic-matrix composites. Compos. Sci. Technol..

[23] Curtin W.A. (1993). Multiple matrix cracking in brittle matrix composites. Acta Metall. Mater..

[24] Hsueh C.H. (1996). Crack-wake interface debonding criterion for fiber-reinforced ceramic composites. Acta Mater..

[25] Gao Y., Mai Y., Cotterell B. (1988). Fracture of fiber-reinforced materials. J. Appl. Math. Phys..

[26] Sun Y.J., Singh R.N. (1998). The generation of multiple matrix cracking and fiber-matrix interfacial debonding in a glass composite. Acta Mater..

[27] Rouby D., Louet N. (2002). The frictional interface: A tribological approach of thermal misfit, surface roughness and sliding velocity effects. Compos. Part A.

[28] Holmes J.W., Cho C.D. (1992). Experimental observation of frictional heating in fiber-reinforced ceramics. J. Am. Ceram. Soc..

[29] Staehler J.M., Mall S., Zawada L.P. (2003). Frequency dependence of high-cycle fatigue behavior of CVI C/SiC at room temperature. Compos. Sci. Technol..

[30] Lee S.S., Stinchcomb W.W. (1994). Damage mechanisms of cross-ply Nicalon/CAS-II laminate under cyclic tension. Ceram. Eng. Sci. Proc..

[31] Lamouroux F., Camus G., Thebault J. (1994). Kinetics and mechanisms of oxidation of 2D woven C/SiC composites: I, experimental approach. J. Am. Ceram. Soc..

[32] Halbig M.C., McGuffin-Cawley J.D., Eckel A.J., Brewer D.N. (2008). Oxidation kinetics and stress effects for the oxidation of continuous carbon fibers within a microcracked C/SiC ceramic matrix composite. J. Am. Ceram. Soc..

[33] Filipuzzi L., Naslain R. (1994). Oxidation mechanisms and kinetics of 1D-SiC/C/SiC composite materials: II, Modelling. J. Am. Ceram. Soc..

[34] Naslain R., Guette A., Rebillat F., Gallet S., Lamouroux F., Filipuzzi L., Louchet C. (2004). Oxidation mechanisms and kinetics of SiC-matrix composites and their constituents. J. Mater. Sci..

[35] Lara-Curzio E. (1999). Analysis of oxidation-assisted stress-rupture of continuous fiber-reinforced ceramic matrix composites at intermediate temperatures. Compos. Part A.

[36] Casas L., Martinez-Esnaola J.M. (2003). Modelling the effect of oxidation on the creep behavior of fiber-reinforced ceramic matrix composites. Acta Mater..

[37] Curtin W.A. (2000). Stress-strain behavior of brittle matrix composites. Comprehensive Composite Materials.

[38] Xia Z.H., Curtin W.A. (2000). Toughness-to-brittle transitions in ceramic-matrix composites with increasing interfacial shear stress. Acta Mater..

[39] Curtin W.A., Ahn B.K., Takeda N. (1998). Modeling brittle and tough stress-strain behavior in unidirectional ceramic matrix composites. Acta Mater..

